# Antenatal corticosteroid administration for foetal lung maturation

**DOI:** 10.12688/f1000research.20550.1

**Published:** 2020-03-30

**Authors:** Katie Wynne, Christopher Rowe, Matthew Delbridge, Brendan Watkins, Karina Brown, Jordan Addley, Andrew Woods, Henry Murray

**Affiliations:** 1 Department of Diabetes & Endocrinology, John Hunter Hospital, New Lambton Heights, NSW, 2305, Australia; 2 Mothers and Babies, Hunter Medical Research Institute, New Lambton Heights, NSW, 2305, Australia; 3 School of Medicine and Public Health, University of Newcastle, Callaghan, NSW, 2308, Australia; 4 School of Medicine and Public Health, University of New England, Armidale, NSW, 2351, Australia; 5 Department of Obstetrics, John Hunter Hospital, New Lambton Heights, NSW, 2305, Australia

**Keywords:** pregnancy, preterm, betamethasone, dexamethasone, respiratory distress syndrome, neonatal mortality, antenatal

## Abstract

Antenatal corticosteroids are an essential component in the management of women at risk for preterm labour. They promote lung maturation and reduce the risk of other preterm neonatal complications. This narrative review discusses the contentious issues and controversies around the optimal use of antenatal corticosteroids and their consequences for both the mother and the neonate. The most recent evidence base is presented.

## Introduction

Preterm birth carries a higher risk of mortality, respiratory distress syndrome (RDS), and other morbidities, particularly prior to 32 weeks’ gestation. Fifty years ago, Professor Sir Graham (Mont) Liggins, a New Zealand obstetrician, first noticed that antenatal corticosteroids (ACS) prevented RDS in premature lambs. Liggins and his paediatric-specialised colleague Howie saw the potential of ACS to reduce the high mortality of preterm babies and undertook a randomised, placebo-controlled trial of ACS in 282 pregnant women anticipated to give birth early ^1^. ACS was delivered as a course of two doses of 12 mg intramuscular betamethasone 24 hours apart. This regimen reduced the rates of neonatal mortality (3% vs. 15%) and RDS (9% vs. 26%). This effect on RDS was significant in babies who were birthed between 2 and 7 days after the first betamethasone dose, and analysis of gestational age showed the reduction in RDS was predominant in the babies born after 26 and before 32 weeks. This landmark paper ignited decades of research and is credited as being one of the greatest innovations in neonatal care.

## Discussion of the literature

Premature birth remains a critical public health issue, with RDS being the primary cause of morbidity and mortality. Glucocorticoids cross the placenta and enhance pulmonary maturation and surfactant production. The 2006 Cochrane systematic review of 21 randomised trials (3,885 women and 4,269 infants) reported that a course of ACS reduced moderate-to-severe RDS (–45%), cerebroventricular haemorrhage (–46%), necrotising enterocolitis (–54%), sepsis in the first 48 hours of life (–44%), and the rate of neonatal death (–31%) ^2^. ACS reduced RDS if administered after 26 and before 35 weeks’ gestation and reduced cerebrovascular haemorrhage and neonatal death if administered after 26 and before 30 weeks’ gestation. There was no significant difference in the rate of chorioamnionitis or puerperal sepsis in treated patients. The authors concluded that “a single course of antenatal corticosteroids should be considered routine for preterm delivery with few exceptions”. A decade later, an updated Cochrane review including 30 randomised controlled studies (7,774 women and 8,158 infants) supported these original conclusions ^3^, although it should be noted that the studies included in these reviews may have been underpowered to detect changes in mortality and the rates of preterm birth were higher than would be expected in contemporary obstetric practice. The evidence for using ACS in the early preterm (EPT: after 25 and before 34 weeks) is substantial, but what about babies born very EPT (VEPT: before 25 weeks), late preterm (LPT: after 34 and before 37 weeks), and term (after 37 weeks)?

### What is the role of antenatal corticosteroids outside early preterm birth?

The efficacy of ACS in VEPT birth (before 25 weeks’ gestation) has been suggested by large cohort studies. Although cohort studies are confounded by factors influencing the clinical decision to administer ACS, randomised trials have been deemed unethical because of the high mortality in this group. A prospective cohort of over 33,000 infants born after 22 and before 26 weeks’ gestation showed ACS increased both survival to hospital discharge (72% vs. 52%) and survival without major morbidity (15% vs. 9%) ^4^. Another prospective cohort of over 10,000 infants exposed to ACS and born after 23 weeks’ gestation showed a reduced composite endpoint of mortality or neurodevelopmental impairment when reviewed in early childhood at 18–22 months ^5^; further analysis showed a reduction in a composite of death and periventricular leukomalacia, intraventricular haemorrhage, or necrotising enterocolitis, and for those born after 22 and before 23 weeks’ gestation a reduction in a composite of death or necrotizing enterocolitis. A separate analysis of 118,000 infants from a US database has shown that the number of women needed to treat to prevent one neonatal death increases exponentially from six at 23–24 weeks’ gestation to 798 at 34 weeks’ gestation ^6^. These three large studies ^4– 6^ were all performed in the United States, and, because they were observational cohorts, the positive effect of ACS may have been confounded by the social inequalities that influence access to healthcare. For example, in two of the studies, mothers administered ACS were more likely to have received prenatal care at all stages of pregnancy and deliver by caesarean section ^4, 6^, whereas in the other study the women not treated with ACS were notably younger, of lower socioeconomic status, and less likely to deliver by caesarean section ^5^. Unfortunately, overall survival without major morbidity remains uncommon in VEPT birth irrespective of ACS treatment. Current guidelines suggest that women giving birth around the time of viability should be considered for ACS ( Table 1).

**Table 1.** Current recommendations for the administration of antenatal corticosteroids in pregnancy.

	RCOG; NICE guidelines	ACOG	RANZCOG (Liggins Institute)	FIGO Working Group on Good Clinical Practice in Maternal–Fetal Medicine	World Health Organization
Title	Preterm labour and birth (1.9.1–1.9.9)	Committee Opinion Number 713, Antenatal Corticosteroid Therapy for Fetal Maturation	Antenatal corticosteroids given to women prior to birth to improve fetal, infant, child, and adult health Clinical Practice Guidelines	Good clinical practice advice: antenatal corticosteroids for fetal lung maturation	WHO recommendations on interventions to improve preterm birth outcomes (1.0–1.10)
Year of publication	2015	2017	2015	2019	2015
Gestational age	24+0 to 33+9 weeks of gestation Consider in patients between 23+0 and 23+6 weeks of gestation and between 34+0 and 35+6 weeks of gestation	24+0 to 36+6 weeks of gestation at risk of birth within 7 days Consider in patients between 23+0 and 23+6 weeks of gestation upon family’s decision	24+0 to 34+6 weeks of gestation Up to 39+0 weeks when lung immaturity known, e.g. amniocentesis for lecithin sphingomyelin	24–34 weeks of gestation <24 weeks of gestation in discussion with family and clinician Consider in patients 34+0 to 36+6 weeks of gestation	24+0 to 34+0 weeks of gestation if there is no evidence of maternal infection and there is available childbirth and neonatal care
Steroid given	Not specified	Betamethasone or dexamethasone	Betamethasone or dexamethasone	Betamethasone or dexamethasone	Betamethasone or dexamethasone
Dosing regimen	Not specified	Betamethasone two 12 mg doses (IM) 24 hours apart OR dexamethasone four 6 mg doses (IM) every 12 hours	Betamethasone 24 mg in divided doses, completed between 12 and 36 hours (as betamethasone sodium phosphate 7.8 mg and betamethasone acetate 6 mg) OR dexamethasone phosphate 24 mg in divided doses completed between 24 and 40 hours	Betamethasone two 12 mg doses (IM) 24 hours apart OR dexamethasone four 6 mg doses (IM) 12 hours apart	Betamethasone (total 24 mg in divided doses) OR dexamethasone (total 24 mg in divided doses)
Recommended dosing interval prior to birth	Not specified	0–7 days prior to birth (2–7 days ideal). Therapy should not be withheld if delivery is anticipated prior to second dose (12 hours after first dose)	0–48 hours prior to birth (up to 7 days), even if birth is likely within 24 hours	Most effective when birth occurs 24 hours after (and up to 7 days) administration of the second dose	Within 7 days of birth, including within 24 hours of birth
Repeat or rescue dosing	Repeat dosing not routinely given. Consider interval since last dose, gestational age, and likelihood of delivering within 48 hours	A single repeat course of ACS when birth is expected within 7 days, <34+0 weeks of gestation, and >14 days since initial dose	A maximum of three, single, repeat doses of ACS when birth is expected within 7 days, <33 weeks of gestation, >7 days since initial dose	A single repeat course of ACS when birth is expected within 7 days, <34+0 weeks of gestation, and >14 days since initial dose	A single repeat course of ACS >7 days from the initial dose when there is high chance of birth in the next 7 days
Prior to elective caesarean section	No recommendation	No recommendation	Recommended for 48 hours if >34+6 weeks of gestation if known fetal lung immaturity	Consider between 37+0 and 38+6 weeks of gestation if clear medical reason for early birth	Not recommended between 34+0 and 36+6 weeks of gestation
Website	https://www.nice.org. uk/guidance/ng25	doi.org/10.1097/ AOG.0000000000002237	https://www.clinicalguidelines. gov.au/portal/2509/antenatal- corticosteriods-given-women- prior-birth-improve-fetal-infant- child-and-adult	doi.org/10.1002/ijgo.12746	https://www.who.int/ reproductivehealth/publications/ maternal_perinatal_health/preterm- birth-guideline/en/

ACOG, American College of Obstetricians and Gynecologists; ACS, antenatal corticosteroids; FIGO, International Federation of Gynecology and Obstetrics; IM, intramuscular; NICE, National Institute for Health and Care Excellence; RANZCOG, Royal Australian and New Zealand College of Obstetricians and Gynaecologists; RCOG, Royal College of Obstetricians and Gynaecologists

LPT babies (born after 34 and before 37 weeks’ gestation) have an increased risk of respiratory and neurodevelopmental complications compared to term babies, but the absolute risk is relatively low. However, because the majority of preterm births are LPT, the total morbidity attributed to LPT birth is substantial. Gyamfi-Bannerman investigated the role of ACS in the Antenatal Late Preterm Steroids (ALPS) trial, a multicentre randomised trial of 2,800 women at high risk of LPT birth ^7^. Betamethasone, given as two 12 mg intramuscular injections 24 hours apart, reduced a composite primary outcome of stillbirth, neonatal death, and the need for respiratory support in the 72 hours after birth (11% vs. 14%), with a number needed to prevent one case of 35. Notably, there were no stillbirths or neonatal deaths in the study and the major benefit of ACS derived from a reduction in transient tachypnoea of the newborn and bronchopulmonary dysplasia (0.1% vs. 0.6%) rather than in RDS. The extremely stringent inclusion criteria, with only 11% of screened women eligible to participate, means this evidence may not be easily translatable to clinical practice. Furthermore, this short-term benefit came at a cost of increased neonatal hypoglycaemia (24% vs. 15%) defined as a glucose level of 40 mg/dl or <2.2 mmol/l, a concern as neonatal hypoglycaemia is an independent risk factor for developmental delay ^8^. It has been suggested that “restricting antenatal steroids for late preterm pregnancies to those expected to deliver at 34–35 weeks could reduce the target population for late preterm antenatal steroids by half (1.6% vs. 4.0% of total deliveries) while still capturing the majority (69%) of newborn respiratory complications in this group” ^9^. Current recommendations for women at risk of LPT birth are inconsistent ( Table 1).

Early term neonates also have a slightly higher risk of RDS if birthed before labour occurs. The ASTECS study randomised almost 1,000 women at 37 weeks’ gestation or beyond to receive betamethasone 48 hours before elective caesarean section. Of the 942 neonates, 24 (5.1%) control babies and 11 (2.4%) treated babies were admitted to intensive care for respiratory distress, including five control babies and one treated baby diagnosed with severe RDS. The number needed to treat to prevent one admission to the neonatal intensive care unit (NICU) was 37 ^10^. Admission to the NICU with respiratory distress fell markedly with increasing gestation at the time of birth, irrespective of exposure, from 37 weeks (5% with ACS exposure vs. 11% without) to 39 weeks’ gestation (0.6% with exposure vs. 1.5% without) and ACS was beneficial at all time points. A slightly larger study of 1,272 women randomised to ACS (three doses of 8 mg intramuscular dexamethasone 8 hours apart, the last 24 hours before caesarean) or standard care prior to a planned caesarean section at 38 weeks’ gestation or beyond did not show a positive effect of ACS ^11^. A subsequent Cochrane review (3,956 women and 3,893 neonates), including these two trials and two smaller studies, concluded that ACS was associated with a reduced risk of RDS (–52%), although the overall risk of RDS was small (1.7%) and quality of evidence was low ^12^. Since this review, another study of early term caesarean section failed to show a benefit of betamethasone on respiratory morbidity ^13^, although the probability of admission to the NICU was reduced (7.2% vs. 2.7%). Disturbingly, expanding the use of ACS to elective caesarean could “increase the population exposed to ACS from about 10–20% of the delivery population to >70%” ^14^.

### What is the role of antenatal corticosteroids in twin pregnancies?

Twin pregnancies are at higher risk of preterm delivery and complications, but these women are often excluded from studies. It has been proposed that ACS may be metabolised differently in twin pregnancies. The levels of betamethasone in cord blood are similar in singleton and twin neonates ^15^, but the half-life may be shorter owing to clearance by two fetoplacental units ^16^. The clinical relevance of this finding is uncertain when the optimal ACS regimen has not yet been established in singleton pregnancy, but it has been conjectured that three doses of 12 mg betamethasone 18 hours apart would be needed to achieve a similar betamethasone profile in twins. A recent large retrospective cohort study of singleton and twin pregnancies birthed after 24 and before 34 weeks’ gestation did show a reduction in neonatal mortality (–58%), RDS (–47%), and severe neurological injury (–50%) in twin preterm babies after completion of a single standard course of ACS (either betamethasone or dexamethasone) 1–7 days before birth, and this positive effect was of a similar magnitude to singleton pregnancies ^17^. Another retrospective cohort study of twin birth has demonstrated a negative effect of ACS exposure in the EPT period on twin birth weight, head circumference, and body length, with a subanalysis showing a dose-dependent effect (betamethasone ≤16 mg vs. 24 mg vs. >24 mg) in the twins born after 34 and before 37 weeks ^18^. This cautions against higher doses of betamethasone, as there was some evidence of harm without benefit. The randomised multicentre ACTWIN study protocol is underway to investigate the effect of standard-dose betamethasone in women at risk of LPT delivery ^19^.

### What is the optimal timing for the administration of antenatal corticosteroids?

The original evidence for the timing of ACS prior to EPT delivery suggested that birth 2–7 days after the first dose may be optimal ^1^. The initial Cochrane review reported a reduction in neonatal death even when infants were born within 24 hours of the first dose, but again no benefit beyond 7 days ^2^. As Gates and Brocklehurst point out, creation of these time categories was arbitrary and, as they have been reproduced in subsequent trials, it is difficult to know what the peak time for administration is and whether it differs according to the choice of steroid or route of administration. The factors that determine whether birth is imminent may also drive responsiveness to ACS, and those babies who are birthed more than 7 days after ACS exposure are apt to be closer to term, when the rates of RDS are considerably lower, making it statistically more difficult to demonstrate any effect ^20^. Pre-clinical data suggest that the effect of ACS may be prolonged beyond 7 days ^14^. Current guidelines reflect the available data that support the administration of ACS in EPT labour when birth is expected within 48 hours, with benefit observed up to 7 days ( Table 1). Further research may elucidate whether this holds true at different gestational ages and birth weights.

### Should repeat course(s) of antenatal corticosteroids be given?

This uncertainty about the durability of ACS has led clinicians to consider a repeat course of ACS if a woman has not birthed within 7 or more days. A Cochrane review of 10 randomised controlled trials (4,733 women and 5,700 babies) showed an incremental short-term benefit of a repeat course(s) of betamethasone on RDS (–17%), with 17 women treated to benefit one baby ^21^. No benefit of a repeat dose was seen for chronic lung disease, intraventricular haemorrhage, or foetal and neonatal mortality. There was a negative effect on head circumference and birth weight, although only the difference in head circumference remained when adjusted for gestational age at birth, and all parameters were similar in the exposed and unexposed groups by hospital discharge. In studies that described early childhood follow-up, there were no differences in growth parameters, but neither were there benefits evident in survival or disability ^21^. This review included the ACTORDS study in which a repeat course of betamethasone (12 mg, 24 hours apart) or dexamethasone (24 mg in 24 hours) was given weekly if the women was still considered likely to birth before 32 weeks’ gestation. This re-stratification of risk generally resulted in a total of two to three ACS courses ^22^. The National Institute of Child Health and Human Development (NICHD) Maternal–Fetal Medicine Units (MFMU) Network study took a slightly different approach. This group enrolled women after 23 and before 32 weeks’ gestation and randomised them to either a single course or weekly courses of betamethasone or dexamethasone administered routinely up to 34 weeks’ gestation, later limited to five courses in total. The latter study was terminated early and showed evidence of harm in women receiving four or more courses of betamethasone, with a higher risk of birth weight below the 10 ^th^ centile (17.3% vs. 8.7%) ^23^. A recent individual-participant data meta-analysis also showed an effect of repeat ACS exposure on birth weight ^24^, and a prospective study of the Finnish birth register demonstrated an effect of ACS treatment on birth weight in 4,887 exposed infants that remained significant irrespective of whether neonates were born preterm (30–34 weeks), near term (35–37 weeks), or term ^25^. The evidence for impaired foetal growth does raise the question of whether ACS are appropriate for growth-restricted babies. Although there is little available evidence for risk or benefit, a recent review suggested that ACS should be used in this group only as part of a clinical trial ^26^. Guidelines differ in their recommendations for repeat courses of ACS ( Table 1).

### Is a risk of neonatal hypoglycaemia important?

When a large number of women require ACS to produce benefit for one baby, it becomes more challenging to estimate the potential overall benefit, as there are, by definition, a greater number of babies exposed unnecessarily. As we have seen, both the ALPS and the ASTECS trials needed to treat over 30 women to prevent one baby requiring respiratory support. Short-term “off-target” effects of treatment include neonatal hypoglycaemia, with a relative risk of 1.6 (24% vs. 15%) after ACS in the ALPS trial ^7^. Maternal hyperglycaemia and consequent neonatal hyperinsulinaemia, or perhaps suppression of the hypothalamic-pituitary-adrenal stress axis, was presumably a substantial contributor to neonatal hypoglycaemia, although maternal glucose levels were not presented despite 10% of the cohort being diagnosed with gestational diabetes. Even women not diagnosed with gestational diabetes may be at risk of maternal hyperglycaemia. A small study has shown that 85% of non-diabetic women develop severe hyperglycaemia (≥160 mg/dl or 8.9 mmol/l) after ACS and all women developed mild hyperglycaemia (fasting >100 mg/dl or 5.6 mmol/l, or post-prandial >120 mg/dl or 6.7 mmol/l) with a consistent peak 3–8 hours after each dose ^27^. Prematurity itself increases the risk of low glucose levels, and guidelines recommend administering ACS close to delivery when maternal hyperglycaemia confers the greatest risk of neonatal hypoglycaemia.

Neonatal hypoglycaemia has been associated with widespread cortical changes on MRI of the developing brain ^28^, visual-motor impairment and executive dysfunction in early childhood, and literacy and numeracy problems in later childhood ^29^. Worryingly, in the CHYLD study, one episode of transient hypoglycaemia (<47 mg/dl or <2.6 mmol/l) was dose-dependently associated with adverse neurodevelopmental outcomes in a prospective cohort of 4.5-year-olds who were born from 32 weeks’ gestation ^30^. The children exposed to hypoglycaemia had a greater risk of a low executive function score and visual motor integration score. Similarly, in a retrospective cohort, neonatal hypoglycaemia within the first 3 hours of life was dose-dependently associated with lower achievement on literacy and mathematics test scores at age 10 years ^31^.

This risk of neonatal hypoglycaemia is particularly relevant to women with gestational or pre-existing diabetes in pregnancy who have an increased chance of preterm birth and may perhaps be more prone to foetal immaturity at a given gestation ^32^. There are few data available for the efficacy of ACS in these women, particularly those with type 1 or type 2 diabetes mellitus, who are often excluded ^2^ owing to the potential risk of foetal acidosis, hyperglycaemia, and stillbirth. Helpfully, the use of an intravenous insulin infusion specifically designed for pregnancy has been shown to reduce maternal hyperglycaemia in women with gestational diabetes after ACS and decrease the incidence of neonatal hypoglycaemia (29% vs. 54%) in babies receiving betamethasone within 48 hours of birth, with a number needed to treat of four ^33^. Diabetes is not a contraindication to ACS, but these evidence-based interventions should be used to limit harm.

### Are there future risks to the child from exposure to antenatal corticosteroids?

The delayed effect of ACS has been evaluated in longer-term randomised trials in which the effect of confounders such as gestational age and comorbidity is minimised. Of particular interest are the modulating effects of ACS on foetal neurodevelopment and the stress response as well as the developmental origins of health and disease ^14^. Endogenous glucocorticoids reduce growth and promote cellular differentiation and are usually at low levels until just before birth. Betamethasone and dexamethasone are more potent agonists of the glucocorticoid receptor and are resistant to enzyme (11β-hydroxysteroid dehydrogenase type-2) degradation in the placenta and foetal brain, resulting in supra-physiological effects. ACS administration in animal studies has been found to alter the myelination of the nervous system, hypothalamic-pituitary-adrenal axis, glucose metabolism, and blood pressure ^34^.

There have now been a number of longer-term follow-up studies of the early randomised efficacy trials that have proven reassuring. The extended original Liggins and Howie randomised cohort (534 of the 988 survivors) were followed-up at age 30 years ^35^. Exposure to ACS as 12 mg betamethasone, two doses 24 hours apart, or for the later cohort double this dose, did not alter growth parameters or cardio-metabolic risk, the only difference being a higher insulin level at 30 minutes after a 75 g glucose tolerance test, suggesting possible mild insulin resistance. A further subset of 192 of these offspring underwent psychological testing, with no difference found in cognitive functioning, working memory and attention, psychiatric morbidity, handedness, or quality of life ^36^. The longer-term effects in babies born under 30 weeks remain unclear, as only 5% of this cohort was born at this gestation.

There has not yet been a longer-term follow-up of the LPT ALPS trial. However, the ASTECS trial of early term caesarean section has reported data from a questionnaire returned by half their 8–15-year-old offspring ^37^. This revealed a possible subtle difference in neurodevelopment: there were no differences in behaviour or standardised tests of academic achievement, but children exposed to ACS were more likely to be in the lower quarter of academic ability as reported by their school (17.7% vs. 8.5%). An effect of ACS on neuropsychological outcomes was also evident in a non-randomised cohort study of term-born children studied at age 6–11 years, who showed increased cortisol reactivity to an acute psychological stress after exposure to a single course of ACS ^38^. A longitudinal cohort study of extremely low-birth-weight infants (<1,000 g) has also suggested an association between ACS exposure and the likelihood of anxiety in adulthood ^39^.

Results from a meta-analysis of three trials with early childhood follow-up of exposure to recurrent doses of ACS reported no difference in neurodevelopment compared to those exposed to a single course ^21^. The ACTORDS study has reported further follow-up of 91% of the children participating in the study of single course or repeated courses of ACS under 32 weeks’ gestation. Despite repeatedly exposed children being more likely to have scores in the clinical range for attention problems at 2 years ^40^, there were no differences in the rate (78% vs. 77%) of survival free of neurosensory disability (including cerebral palsy), body size, or general health at 6–8 years ^41^. Neither was there evidence of a difference in body composition, insulin sensitivity, blood pressure ^42^, or bone mass in a subset of the same children ^43^. The NICHD-MFMU Network study of routinely repeated ACS doses in women at risk of EPT birth, included in the meta-analysis, concluded that repeated courses of ACS may contribute to neurodevelopmental differences observed in early childhood at 2–3 years. This study showed a trend toward an increase in cerebral palsy in those exposed to multiple courses (2.9%, n = 6 vs. 0.5%, n = 1; *P* = 0.12). There were six babies diagnosed with mild cerebral palsy born to women who received four to five total courses of ACS compared to one baby born with severe cerebral palsy after single course exposure. Five of these six babies were born at or after 34 weeks’ gestation, when cerebral palsy is usually less common. Perhaps this is a real effect, as randomised studies evaluating postnatal treatment of pre-term infants with glucocorticoids have also showed an increased risk of cerebral palsy ^44^.

### What is the optimal antenatal corticosteroid regimen?

There are many unanswered questions about the optimal choice of ACS steroid, dose, and regimen, leading to considerable variation in practice with differences in the use of betamethasone (phosphate and acetate) and dexamethasone (phosphate) between countries. The World Health Organization (WHO) have identified that the investigation of potential differences between these preparations is a research priority. Both glucocorticoids provided comparable short-term benefits in a Cochrane review of seven studies using dexamethasone (1,585 women and 1,798 infants) and 21 studies using betamethasone (6,133 women and 6,134 infants) ^3^. The standard dosing regimen is based on equivalence dosing from the original pre-clinical studies and has not been further optimised to minimise foetal exposure ^14^. Interestingly, a recent study in sheep suggested that it is the duration of exposure to low-dose ACS, rather than total exposure or peak level, which mediates lung maturation ^45^. This study also suggested potential sex-linked differences in response to ACS that warrant further investigation. Perhaps ACS should be weight-adjusted, as it may cause more severe prolonged maternal hyperglycaemia in women with lower body mass index ^46^. Two studies in progress may address some unknowns: the A*STEROID study will randomise women to receive either betamethasone or dexamethasone before 34 weeks’ gestation to determine if there is a differential effect on neurosensory ability in their children at 2 years of age ^47^, and the BETADOSE non-inferiority study will examine whether one or two doses of betamethasone are needed in a course of ACS treatment for women receiving a first dose before 32 weeks’ gestation ^34^.

### What about women with preterm premature rupture of membranes?

Data from the most recent Cochrane review demonstrated a reduction in neonatal death, RDS, and necrotising enterocolitis in the infants of mothers who received ACS for preterm premature rupture of membranes (PPROM) ^3^. Although there is evidence of benefit, concerns have arisen about potential harm from ACS triggered by a secondary analysis of data that demonstrated an increased risk of chorioamnionitis in women with PPROM who received repeated weekly courses of steroid, rather than a single ACS treatment ^48^. Recent larger studies have suggested no increased risk of chorioamnionitis or neonatal sepsis with ACS use, even if one repeat course was given ^49, 50^. A meta-analysis of seven observational studies demonstrated that ACS (including repeat treatments) was safe and effective in EPT infants with subclinical chorioamnionitis, although when chorioamnionitis was clinically apparent the benefits became less obvious ^51^. It has been postulated that chorioamnionitis may alter endogenous corticosteroid exposure in the foetus, perhaps diminishing the magnitude of the effect of exogenous steroid. The available literature supports administration of ACS in women with PPROM to reduce morbidity and mortality in EPT labour in the absence of overt infection.

### Are women optimally selected for antenatal corticosteroids?

It is common for babies treated with ACS not to be delivered prematurely as anticipated. Indeed, in the Finnish birth register study of exposure to ACS, the mean gestation at birth was 35 (±4) weeks and 44% of women delivered after 37 weeks’ gestation ^25^. A Canadian study of 250,000 births showed that from 1998–2012, the proportion of women receiving optimal ACS treatment given between 24 and 34 weeks’ gestation within 24 hours to 7 days of birth increased (10% vs. 23%), but this was accompanied by greater ACS exposure after 34 weeks’ gestation (0.2% vs. 1.7%) and a concurrent increase in the proportion of treated women at risk of EPT delivery who did not give birth within the optimal time window (7% vs. 34%) ^52^. The challenge of predicting imminent preterm delivery increases the risk of unnecessary ACS treatment; indeed, in this study, 3.2% of all births from 2008–2012 were exposed to ACS treatment and 52% of these women delivered at 35 weeks or later ^52^. A more recent retrospective Swedish study of 500 EPT neonates born from 2013–2016 also showed 29% were born outside the optimal window more than 7 days after the administration of ACS ^53^. An Australian study from 2014–2015 reported that 9.6% of all women in one centre received ACS, and less than one-third received appropriately timed treatment ^54^. This is of concern not just from the perspective of a loss of treatment efficacy but also as a potential cause of harm, with a retrospective cohort study suggesting an increase in perinatal mortality if ACS treatment was administered more than 7 days prior to delivery ^55^. Better predictive models are needed to provide a more accurate probability of the timing of delivery ^52^. To reduce the significant burden of “off-target” adverse effect for infants treated unnecessarily or inappropriately, the International Federation of Gynecology and Obstetrics (FIGO) guidelines recommend that cervical length and fibronectin/PAMG1 measurements should be considered in women to better assess the likelihood of preterm birth ^56^.

There remains a potential for harm using ACS for extended indications. The large cluster-randomised Antenatal Corticosteroids Trial (ACT) assessed the risks and benefits of ACS use in women anticipated to deliver at 24–36 weeks’ gestation in lower resource rural and semi-urban settings in Argentina, Guatemala, India, Kenya, Pakistan, and Zambia ^57^. A multifaceted intervention was designed to improve the recognition of preterm birth and promote the use of a single dexamethasone course. The 28-day neonatal mortality in infants born under the fifth birth centile (a proxy for EPT or LPT infants) was not lower (225 per 1,000 live births) in the 2,520 infants within the intervention clusters (45% exposed to ACS) than the mortality (232 per 1,000 live births) in 2,258 infants in the control clusters (10% exposed to ACS); suspected maternal infection was significantly higher in the intervention clusters (10% vs. 6%). Perhaps most alarming was the data for the whole group, including all birth weights, that showed an increased risk of both neonatal mortality (relative risk 1.12) and suspected maternal infections (odds ratio 1.45) in the intervention clusters (47,394 livebirths), of whom 12% received ACS compared with 2% in the control clusters (50,743 livebirths). The authors conjectured that the failure to demonstrate a benefit from ACS in infants born under the fifth centile may have been due to the unavailability of neonatal intensive care and that overtreatment with ACS in the intervention clusters could have caused harm in this context, as only 16% of these women went on to deliver an infant under the fifth birth centile.

## Conclusions

Evidence accumulated after the Liggins and Howie study ^1^ has confirmed that ACS treatment reduces neonatal morbidity and mortality if appropriately administered in the 7 days before delivery in selected women at high risk of EPT birth, particularly before 30 weeks’ gestation. Trial data have shown that the rate of RDS in preterm babies can be almost halved, with persuasive evidence for an overall respiratory benefit in the EPT group in whom the absolute risk of RDS is higher. Although most of these data were collected prior to the advent of contemporary innovations such as magnesium, non-invasive ventilation, and surfactant, the 2017 Cochrane review has indicated that further randomised trials are not warranted ^3^. Cohort studies of VEPT birth support the administration of ACS, and randomised trials are unlikely to be conducted. The studies of ACS in LPT and term caesarean birth show marginal benefits in self-limiting respiratory complications; therefore, the potential for short- and longer-term harm should be carefully balanced with these women. The risk of ACS exposure is greater after four or more courses of ACS, with some evidence of at least transient neonatal growth limitation and later subtle neurodevelopmental differences with changes to the hypothalamic-pituitary-adrenal stress axis.

The use of ACS delivered optimally in high resource settings saves lives and is a revolution in the care of preterm neonates. For this reason, ACS is recommended for EPT labour by the Royal College of Obstetricians and Gynaecologists (RCOG), National Institute for Health and Care Excellence (NICE), American College of Obstetricians and Gynecologists (ACOG), Royal Australian and New Zealand College of Obstetricians and Gynaecologists (RANZCOG), FIGO, and WHO; ACS can be considered in LPT labour as suggested by the RCOG, NICE, RANZCOG, and FIGO guidelines. Further research is needed to establish efficacy in diabetes, growth restriction, and twin pregnancy. It remains a challenge to optimise ACS use and explain the risks and benefits of ACS accurately to empower shared decision-making where the evidence is uncertain.
